# A widespread picornavirus affects the hemocytes of the noble pen shell (*Pinna nobilis*), leading to its immunosuppression

**DOI:** 10.3389/fvets.2023.1273521

**Published:** 2023-12-13

**Authors:** Francesca Carella, Patricia Prado, Gionata De Vico, Dušan Palić, Grazia Villari, José Rafael García-March, José Tena-Medialdea, Emilio Cortés Melendreras, Francisca Giménez-Casalduero, Marco Sigovini, Serena Aceto

**Affiliations:** ^1^Department of Biology, University of Naples Federico II, Naples, Italy; ^2^Institute of Agrifood Research and Technology (IRTA)-Sant Carles de la Ràpita, Tarragona, Spain; ^3^Chair for Fish Diseases and Fisheries Biology, Faculty of Veterinary Medicine, Ludwig-Maximilians-University Munich, Munich, Germany; ^4^Instituto de Investigación en Medio Ambiente y Ciencia Marina, Universidad Católica de Valencia, Calpe, Spain; ^5^Murcia University Aquarium, University of Murcia, Murcia, Spain; ^6^Department of Marine Science and Applied Biology, Research Marine Centre in Santa Pola (CIMAR), University of Alicante, Alicante, Spain; ^7^Consiglio Nazionale delle Ricerche, Istituto di Scienze Marine, Venice, Italy

**Keywords:** immunosuppression, mass mortality, noble pen shell, RT-PCR, RNA virus

## Abstract

**Introduction:**

The widespread mass mortality of the noble pen shell (*Pinna nobilis*) has occurred in several Mediterranean countries in the past 7 years. Single-stranded RNA viruses affecting immune cells and leading to immune dysfunction have been widely reported in human and animal species. Here, we present data linking *P. nobilis* mass mortality events (MMEs) to hemocyte picornavirus (PV) infection. This study was performed on specimens from wild and captive populations.

**Methods:**

We sampled *P. nobilis* from two regions of Spain [Catalonia (24 animals) and Murcia (four animals)] and one region in Italy [Venice (6 animals)]. Each of them were analyzed using transmission electron microscopy (TEM) to describe the morphology and self-assembly of virions. Illumina sequencing coupled to qPCR was performed to describe the identified virus and part of its genome.

**Results and discussion:**

In 100% of our samples, ultrastructure revealed the presence of a virus (20 nm diameter) capable of replicating within granulocytes and hyalinocytes, leading to the accumulation of complex vesicles of different dimensions within the cytoplasm. As the PV infection progressed, dead hemocytes, infectious exosomes, and budding of extracellular vesicles were visible, along with endocytic vesicles entering other cells. The THC (total hemocyte count) values observed in both captive (eight animals) (3.5 × 10^4^–1.60 × 10^5^ ml^−1^ cells) and wild animals (14 samples) (1.90–2.42 × 105 ml^−1^ cells) were lower than those reported before MMEs. Sequencing of *P. nobilis* (six animals) hemocyte cDNA libraries revealed the presence of two main sequences of *Picornavirales*, family *Marnaviridae*. The highest number of reads belonged to animals that exhibited active replication phases and abundant viral particles from transmission electron microscopy (TEM) observations. These sequences correspond to the genus *Sogarnavirus*—a picornavirus identified in the marine diatom *Chaetoceros tenuissimus* (named *C. tenuissimus* RNA virus type II). Real-time PCR performed on the two most abundant RNA viruses previously identified by *in silico* analysis revealed positive results only for sequences similar to the *C. tenuissimus* RNA virus. These results may not conclusively identify picornavirus in noble pen shell hemocytes; therefore, further study is required. Our findings suggest that picornavirus infection likely causes immunosuppression, making individuals prone to opportunistic infections, which is a potential cause for the MMEs observed in the Mediterranean.

## 1 Introduction

Since June 2016, thousands of the noble pen shell *Pinna nobilis* individuals have died in the Mediterranean due to an extensive mass mortality event (MME) (1–7). The wide geographic range of the phenomenon makes the mass mortality of the species one of the largest known marine wildlife epizootic mortality events to date in the Mediterranean Sea (2, 8). The event was first observed along hundreds of kilometers of the southeastern coast of the Iberian Peninsula (9). Mass mortalities were subsequently observed in the northwestern Mediterranean (French and Italian coasts) and soon after in Greece, Cyprus, Turkey, Algeria, Tunisia, Morocco, Albania, and Croatia (1–5). The cause of this MME has been attributed to different pathogens, particularly *Haplosporidium pinnae* and *Mycobacterium* spp. (10–12). The presence of a great variety of pathogens associated with MMEs, including several potentially opportunistic pathogens, suggests that disease pathogenesis leading to animal mortality may have other unidentified causes (4, 13).

The order *Picornavirales* comprises positive-strand RNA viruses ranging between 7,000 and 12,500 nt in length (14). Within this order, the family *Picornaviridae* comprises 29 genera (https://ictv.global/report/chapter/picornaviridae/picornaviridae) of picornaviruses (PVs) (15). Picornaviruses are small, icosahedral viruses with single-stranded, highly diverse positive-sense RNA genomes associated with mild to severe diseases in vertebrates, invertebrates, and plants (16–18). The family *Picornaviridae* includes some of the most important groups in the development of virology, comprising poliovirus, rhinovirus, and hepatitis A virus (18, 19).

Virions consist of a capsid with no envelope surrounding a core of ssRNA measuring 22–33 nm in diameter (14, 20, 21). Next-generation sequencing (NGS) has significantly expanded the order *Picornavirales* in recent years through the identification of previously unknown viruses associated with various taxa, including aquatic vertebrates and invertebrates (22–27). The replication cycle takes place in the cytosol in close association with reorganized cytoplasmic membranous structures (28, 29). Infection with PV can induce numerous changes in infected cells, with the massive accumulation of cytosolic double-membraned vesicles (DMVs) and replication organelles possibly being the most important changes (30–32). Picornaviruses target intracellular membranes to generate complex membrane rearrangements of host organelles, such as the endoplasmic reticulum (ER), mitochondria, or endolysosomes (33, 34). Host intracellular membranes contain molecules, lipids, and proteins that serve as vehicles for intercellular communication in various (patho)physiological processes (35). The membrane may provide optimal platforms for viral RNA synthesis by concentrating viral replicative proteins and relevant host factors, as well as hiding replication intermediates, contributing to the evasion of host innate immune sensors (36).

Here, we report the discovery of a previously undescribed picornavirus infecting the immune cells of the noble pen shell *Pinna nobilis* sampled between 2021 and 2023 in different regions of Spain and Italy, where mortality events have been reported. Thirty specimens were analyzed using transmission electron microscopy (TEM) to describe the morphology and self-assembly of virions within the hemocyte cytoplasm and major structural transformations occurring in infected host cells. Illumina sequencing coupled to qPCR was performed to describe the identified virus and part of its genome.

## 2 Materials and methods

### 2.1 Hemolymph collection and investigation of viral agents in hemocytes

**Figure 1:**
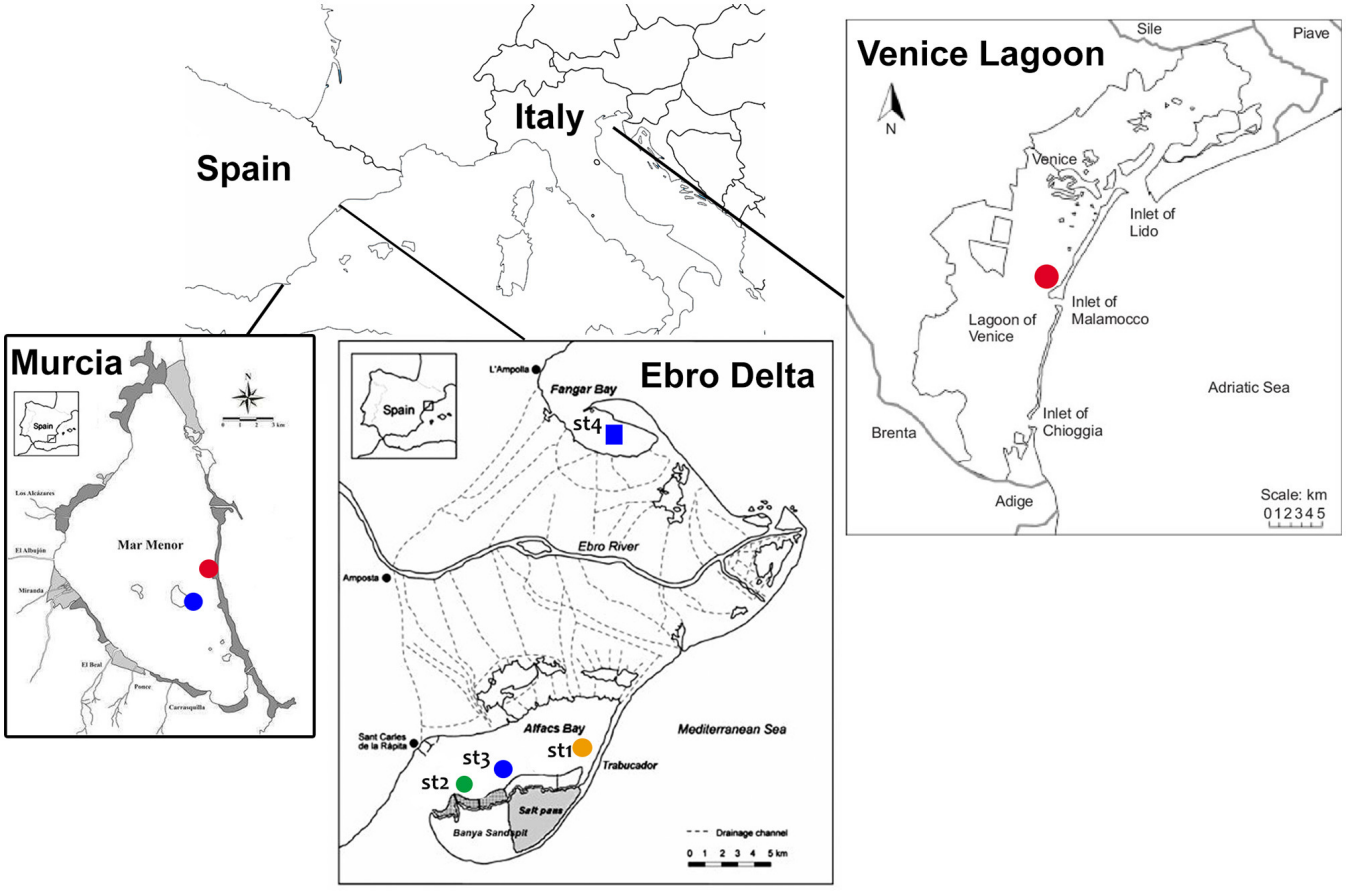
Map of the study areas where noble pen shell *Pinna nobilis* hemocytes were sampled in 2021–2023 in Murcia Baron (in blue), La Manga (in red), the Ebro Delta (sites 1–4), and the Venice Lagoon (Ottagono Alberoni).

Due to its status as an endangered species, sampling of *P. nobilis* was carried out with the permission of regional and national authorities for animal welfare (for Spain, Generalitat de Catalunya—Identificació de l'expedient: SF/0003/23; Bank of species of the Mar Menor INF/2020/0017 promoted by the General Directorate of the Mar Menor and the University of Murcia; for Italy, Prot. MATTM 0016478 05/03/2020). A total of 30 animals were included in the study, collected from July 2021 to May 2023. Sampling was performed on natural populations along the Mediterranean coasts of Italy (*n* = 4) and Spain (*n* = 18) and on animals maintained in captivity in two facilities in Spain (*n* = 8). The natural sites in Spain and Italy were selected according to the presence of remaining populations of *P. nobilis*, despite the occurrence of mortality outbreaks (Figure 1).

In Catalonia (Spain), samples were collected from two estuarine populations in the Ebro Delta, where residual populations still exist: Fangar Bay (northern hemidelta) and Alfacs Bay (southern hemidelta), which are distributed in four different monitored areas (28). Mortality was first observed in 2018, leaving only a few individuals by 2021 (37). The mortality rate progressively increased from 33.5% in September 2021 to 75% in June 2023 (Prado pers. comm). Some of the remaining animals from this area are maintained in the aquarium facilities of the Instituto de Investigación en Medio Ambiente y Ciencia Marina (IMEDMAR) of the Universidad Católica de Valencia (UCV), in Valencia, Spain, and were included in the study. Another remaining population in Spain is the one located at the Mar Menor lagoon (Murcia), in the southwestern part of the Mediterranean. In this area, *P. nobilis* mortality was first observed in the summer of 2016. Initially, the *P. nobilis* mortality was correlated with an environmental collapse that became critical in 2016, 2019, and 2021 and reduced the population by >99% (38); for the present work, two surviving localities were sampled in the Baron and La Manga areas (39); some animals (n = 2) from this area were transferred to the seawater tanks of the University of Murcia Aquarium. Animals (*n* = 6) had been kept in captivity at IMEDMAR and Murcia Aquarium facilities for 6 months to 1 year. Until recently, *P. nobilis* was widely distributed in the Venice Lagoon (Italy), with the largest and densest colonies over marine tidal deltas and the outer part of central basins (40). Mortality onset was observed at the end of 2020, when the population decreased between 60 and 80% (unpublished data). The animals (*n* = 4) were sampled near Ottagono Aberoni Island, approximately 1 km away from the Malamocco inlet (Sigovini, personal communication).

For each animal included in the study (from the field and in captivity), 2 ml of hemolymph was collected via a 23-gauge needle from the posterior adductor muscle as a non-destructive sampling. The presence of clinical signs of disease was considered during sampling. Previous reports in the literature indicate that outward signs of disease start with behavioral changes, including shell gaping, slow valve closure speed when touched, and mantle retraction into the valves from the edge of the shell (11). These signs were difficult to recognize in animals in the field and those maintained in captivity since they mostly appear at very late stages of disease (11). In Catalonia, on July 21 and July/August 2022, most of the animals in the study (19) displayed apparent slow valve closure. In Venice Lagoon, in May 2023, animals displayed quick valve closure and visible mantle at the border of the valves instead. Nevertheless, 2 months later, remarkable mortality was observed in the sampling area. Animals maintained in the Murcia Aquarium and IMEDMAR-UCV did not show apparent signs of disease, but in both cases, up to 40–60% of the animals suffered sudden mortality in the following 6 months, primarily attributed to manipulation from artificial spawning attempts.

After the procedure, the animals were immediately relocated to the seabed/tanks, and their health was monitored over the following days. A volume of ~2 ml of hemolymph was collected for each animal. A 1 ml hemolymph aliquot was placed in a 1 ml RNA Later (Sigma-Aldrich, Italy)-labeled tube placed on ice for molecular analyses, and the other half was preserved for ultrastructural analyses. A small hemolymph aliquot (20 μl) was placed on a hemocytometer (Burker chamber) to determine the total cell number ( × 10^5^/ml) and define the total hemocyte count (THC) (Table 1).

**Table 1:** List of collected samples of noble pen shell *P. nobilis* hemolymph in the Mediterranean Sea.

**Site**	**Month and year**	**Number of collected animals evaluated by TEM**	**Sample type**	**Mean THC (ml^−1^) of collected animals over the years**
Alfacs site 1	July 2021	6	Hemolymph	(4 in 2021): 2.35 ± 0.82 cells × 10^5^
Trabucador	July 2022			(2 in 2022): 1.90 ± 0.33 cells × 10^5^
Alfacs site 2	July 2021	5	Hemolymph	(3 in 2021): 2.42 ± 0.42 cells × 10^5^
Xiringuito	July 2022			(2 in 2022): 2.15 ± 0.34 cells × 10^5^
Alfacs site 3	July 2021	4	Hemolymph	(3 in 2021) 2.30 ± 1.0 cells × 10^5^
Barca	August 2022			2022 n.a.
Fangar site 4	May 2023	3	Hemolymph	n.a.
Murcia aquarium	September 22	4	Hemolymph	1.60 ± 0.22 cells × 10^5^
Tanks of IMEDMAR-UCV	July 21	6	Hemolymph	(4 in 2021): 7.9 ± 0.3 cells × 10^4^
	May 2023			(2 in 2023) 3.5 ± 0.3 cells × 10^4^
Venice lagoon	May 2023	6	Hemolymph	n.a.

The number of samples analyzed per year is indicated in parentheses.

n.a., data not available; THC, total hemocyte count.

### 2.2 Transmission electron microscopy

For all the animals included in the study, part of the hemolymph was also fixed in 2.5% glutaraldehyde in PBS for 3 h. Cells were postfixed in 1% osmium tetroxide for 60 min and 0.25% uranyl acetate overnight. The cells were then dehydrated through a graded ethanol series and embedded in EPON resin overnight at 37°C, 1 day at 45°C, and 1 day at 60°C. Ultrathin sections (80 nm) were cut parallel to the substrate and placed onto a 200-mesh copper grid. Bright-field TEM images were obtained on the dried sample by using an FEI TECNAI G2 200 kV s-twin microscope operating at 120 kV (Thermo Fisher Scientific, Waltham, USA). Digital images were acquired with an Olympus VELETA camera.

### 2.3 RNA extraction, sequencing, and bioinformatic analysis

Total RNA was extracted from the hemocytes of six samples of *P. nobilis* collected from three sites of the wild population in Alfacs Bay, Catalonia (Table 2) using the PureLink RNA Mini Kit (Thermo Fisher Scientific, Waltham, Massachusetts, USA). Following DNase treatment, RNA concentration and quality were measured using a Nanodrop 2000 spectrophotometer (Thermo Fisher Scientific). Strand-specific cDNA libraries were prepared, and Illumina sequencing (NovaSeq 6000, 150 bp paired-end) was carried out by Eurofins Genomics (Ebersberg, Germany). Raw reads underwent trimming and adapter clipping using Trimmomatic 0.4 (41).

**Table 2:** Samples of the noble pen shell *P. nobilis* used to extract RNA from hemocytes and raw data sequencing statistics.

**Sample ID**	**Collection place**	**Collection date**	**Sequenced reads**	**Sequenced bases**
Bar1	Alfacs Bay site 3	August 2022	25,189,100	7,556,730,000
Bar2	Alfacs Bay site 3	August 2022	28,331,700	8,499,510,000
Trab8a	Alfacs Bay site 1	July 2022	27,304,600	8,191,380,000
Trab8b	Alfacs Bay site 1	July 2022	29,977,200	8,993,160,000
Xir2a	Alfacs Bay site 2	July 2022	27,649,600	8,294,880,000
Xir5a	Alfacs Bay site 2	July 2022	23,687,100	7,106,130,000

To identify RNA viruses eventually infecting the *P. nobilis* hemocytes, the trimmed, good-quality reads were aligned to the *P. nobilis* genome (ASM1616189v1) using Bowtie2 (42), and the unmapped reads were extracted using SAMtools (43).

A total of 33,097,808 paired unmapped reads were assembled using Trinity 2.12.0 (44), and the abundance estimation of contigs was performed with RSEM (45).

The Trinity-assembled contigs (140,922) were used as queries in a BLASTX search against the viral protein database (https://ftp.ncbi.nlm.nih.gov/refseq/release/viral/, downloaded on 6 June 2023). To focus the annotation on RNA viruses, the assembled contigs were analyzed using Virsorter2 (46) by selecting only the classification of RNA viruses. Additionally, VirBot, an RNA viral contig detector for metagenomic data (47), was utilized with the sensitive option. The contigs identified as positive by both VirSorter2 and VirBot were subjected to analysis using ORFfinder (https://www.ncbi.nlm.nih.gov/orffinder/). The resulting ORFs were then subjected to a BLASTP search against the UniProtKB/Swissprot, RefSeq protein, and non-redundant protein sequence databases on 4 July 2023.

The predicted sequences of the RNA-dependent RNA polymerase (RdRp) protein encoded by the identified RNA viruses were used for BLASTP searches against the viral protein database, and the highest scoring homologous sequences were downloaded. Multiple amino acid alignments of the selected RdRp sequences were performed using the Constraint-based Multiple Alignment Tool (COBALT, https://www.ncbi.nlm.nih.gov/tools/cobalt/re_cobalt.cgi). The best amino acid substitution model was searched, and the maximum likelihood tree was generated using the LG + G + I model and 500 bootstrap replicates using MEGAX (48), resulting in a final dataset of 317 characters.

### 2.4 Quantitative real-time PCR to detect the presence of the virus in *Pinna* hemocytes

Total RNA was extracted from the hemocytes of other 5 animals *P. nobilis* samples collected from different areas in Alfacs Bay, Catalonia (Table 3), as described before. RNA (500 ng) was reverse-transcribed using Maxima First Strand cDNA Synthesis (Thermo Scientific). Specific primer pairs were designed to amplify the two most abundant RNA viruses identified by the *in silico* analysis (Table 4), and quantitative real-time PCR experiments were conducted. Amplification reactions were performed in a total volume of 10 μl using 5 μl of PowerUp SYBR Green Master Mix (Applied Biosystems), 0.2 μM of each specific primer, and adjusted to 10 μl with distilled water. The reactions were conducted in technical duplicates using *P. nobilis* elongation factor-1 (*EF-1*) as a reporter gene (49, 50) (Table 4). The amplification conditions were as follows: 1 cycle for 2 min at 50°C; 1 cycle for 2 min at 95°C; 40 cycles of amplification at 95°C for 15 s; 60°C for 18 s; and 72°C for 1 min. The reactions were conducted in the QuantStudio 3 Real-Time PCR System (Thermo Scientific). The cDNA of the samples identified as Trab8a and XIR2a (the same used in the RNA-seq experiment) was subjected to real-time PCR amplification to confirm the results of the *in silico* analysis, and two negative controls were run without cDNA.

**Table 3:** Samples of the noble pen shell *P. nobilis* used to extract RNA from hemocytes used for qPCR.

**Sample ID**	**Collection place**	**Collection date**
Bar6	Alfacs Bay site 3	August 2022
Bar7	Alfacs Bay site 3	August 2022
Trab3	Alfacs Bay site 1	July 2022
Trab5	Alfacs Bay site 1	July 2022
Xir6	Alfacs Bay site 2	July 2022

**Table 4:** Primers used for the assessment of virus presence in noble pen shell *Pinna nobilis* hemocytes.

**Primer**	**Sequence**	**Amplicon length (bp)**	**Reference seq accession number**	**Primer position on reference seq**
**PNChetoF**	**AGGGATGTTTGTGGGAGCAC**	**193**	**OR448788**	**5,561–5,580**
**PNChetoR**	**ACCGCCGAGGGTATTCTACT**			**5,753–5,734**
PNPicornF	CGTCCGATGCCTCTCACTAC	167	OR448789	5,702–5,721
PNPicornR	AACCGGTCGAGCCAAGAAAT			5,868–5,849
EF1bivF	CTGGGTKTTGGACAAACTGAAG	211	XM_034472995.1	299–320
EF1bivR	GATACCAGCTTCAAATTCACCA			509–488

## 3 Results

### 3.1 Total hemocyte count, ultrastructure of infected hemocytes, and virus details

**Figure 2:**
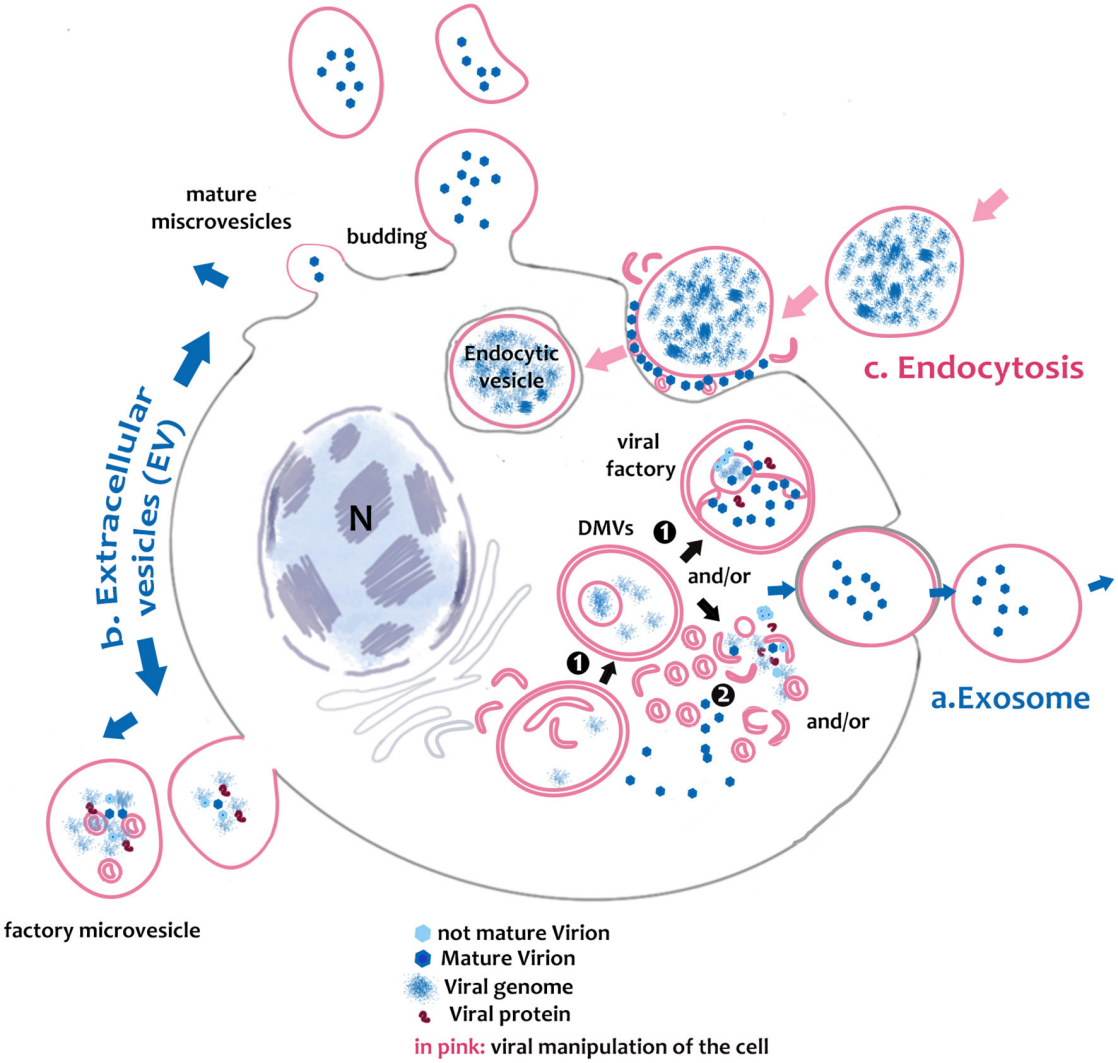
Schematic of picornavirus spread in noble pen shell *Pinna nobilis* hemocytes. Virus can form in large DMVs (1) or in small microvesicles (2), where the virus is freed in the cytoplasm. Then, viral replication and release may be performed using different pathways. a. Viruses may use the autophagic-like vesicles for viral spread, packaging virions, or viral factors that will be released to the extracellular medium as exosomes after fusion with the plasma membrane; b. Infectious microvesicle-like structures can either bud as evagination directly from the cytoplasm containing assembled virions and exit in extracellular vesicles (EVs) with mature virions or as factory microvesicles; then c. PV infective mechanism involves vesicles entering through the endocytic pathway.

**Figure 3:**
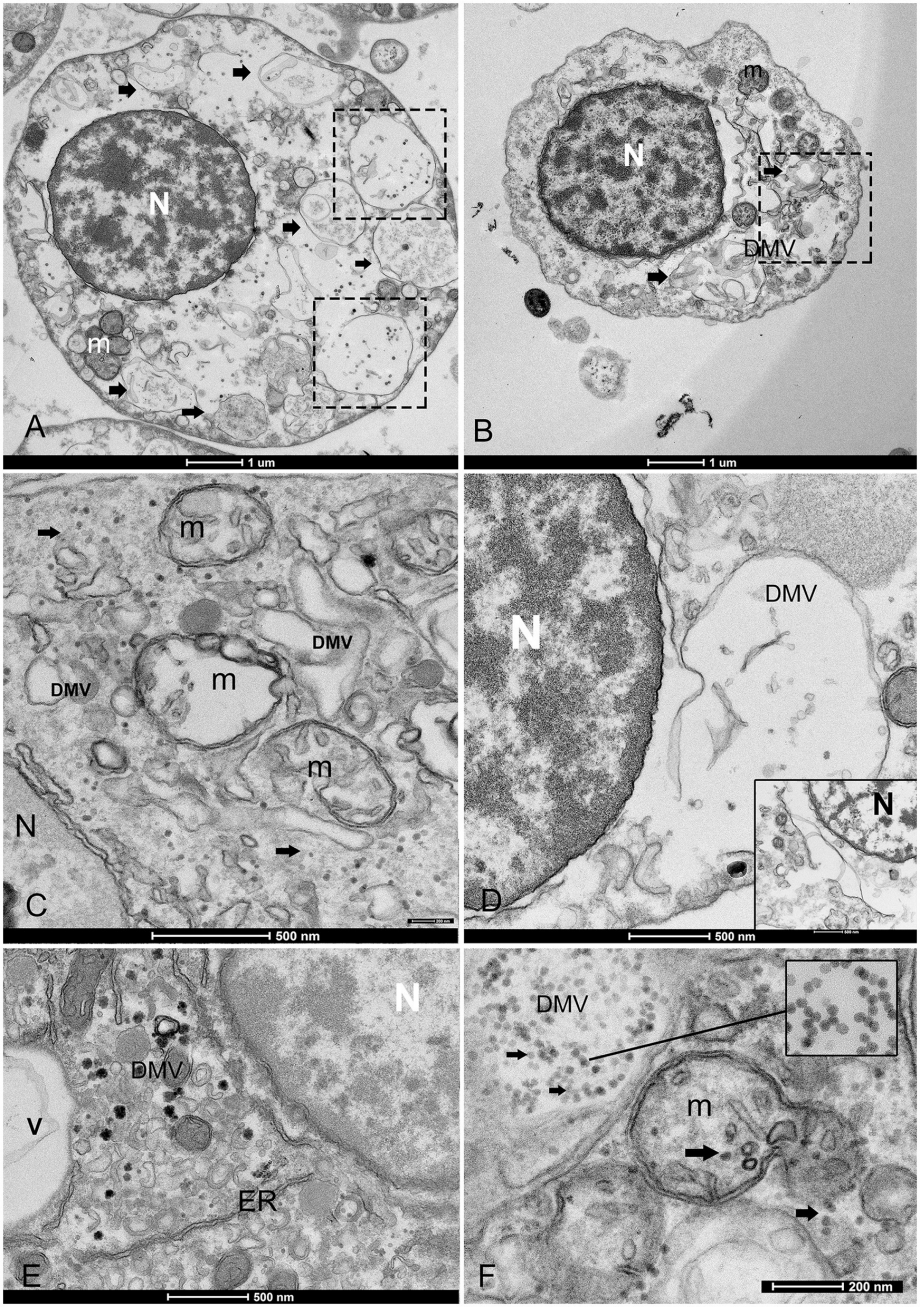
Overview of the morphological features of viral infection in the hemocytes of noble pen shell *Pinna nobilis*. **(A, B)** Granulocytes **(A)** and hyalinocytes **(B)** displaying double-membrane vesicles (DMVs) (arrows and squares). **(C)** Small vesicles (100–200 nm DMVs) and damaged mitochondria (m) with visible presence of viral particles in the cytoplasm (arrows); **(D)** Vesicle originates from the ER and nuclear membrane (inset); **(E)** Endoplasmic reticulum (ER) rearranged, forming DMV (v) and detail of zippered ER that consists of long stretches of ER-derived paired membranes. **(F)** Detail of vesicles containing viral particles (inset) and virus replication in a mitochondrion (m) but also present in the cell free in the cytoplasm (arrows). *N*, nucleus.

The THC values varied among individuals and areas over the years. The mean hemocyte count showed the lowest values from animals maintained in captivity in the IMEDMAR-UCV and Murcia Aquarium (between 3.5 × 10^4^ and 1.60 × 10^5^ ml^−1^ cells) compared with those from the natural population in Catalonia (1.90–2.42 × 10^5^ ml^−1^ cells) (Table 1). The most represented cells were granulocytes (~10 μm), displaying a small peripheral nucleus, and a small quantity of hyalinocytes (~7 μm), presenting a large central nucleus and small cytoplasm. All the hemocytes collected from animals in all areas of Italy and Spain from 2021 to 2023 (100%) displayed ultrastructural features of viral infection with associated cell death. These hemocytes all exhibited complex and unique membrane relocations, displaying numerous cytoplasmic vesicles associated with damaged mitochondria, a total lack of granules, the presence of protein aggregates, and, in some cases, cellular buddings. The formation of invaginations occurred at the membrane of various organelles, including the ER, endolysosomes, and mitochondria (Figures 2, 3). Features of viral infection at the hemocyte level are represented in Figure 2.

**Figure 4:**
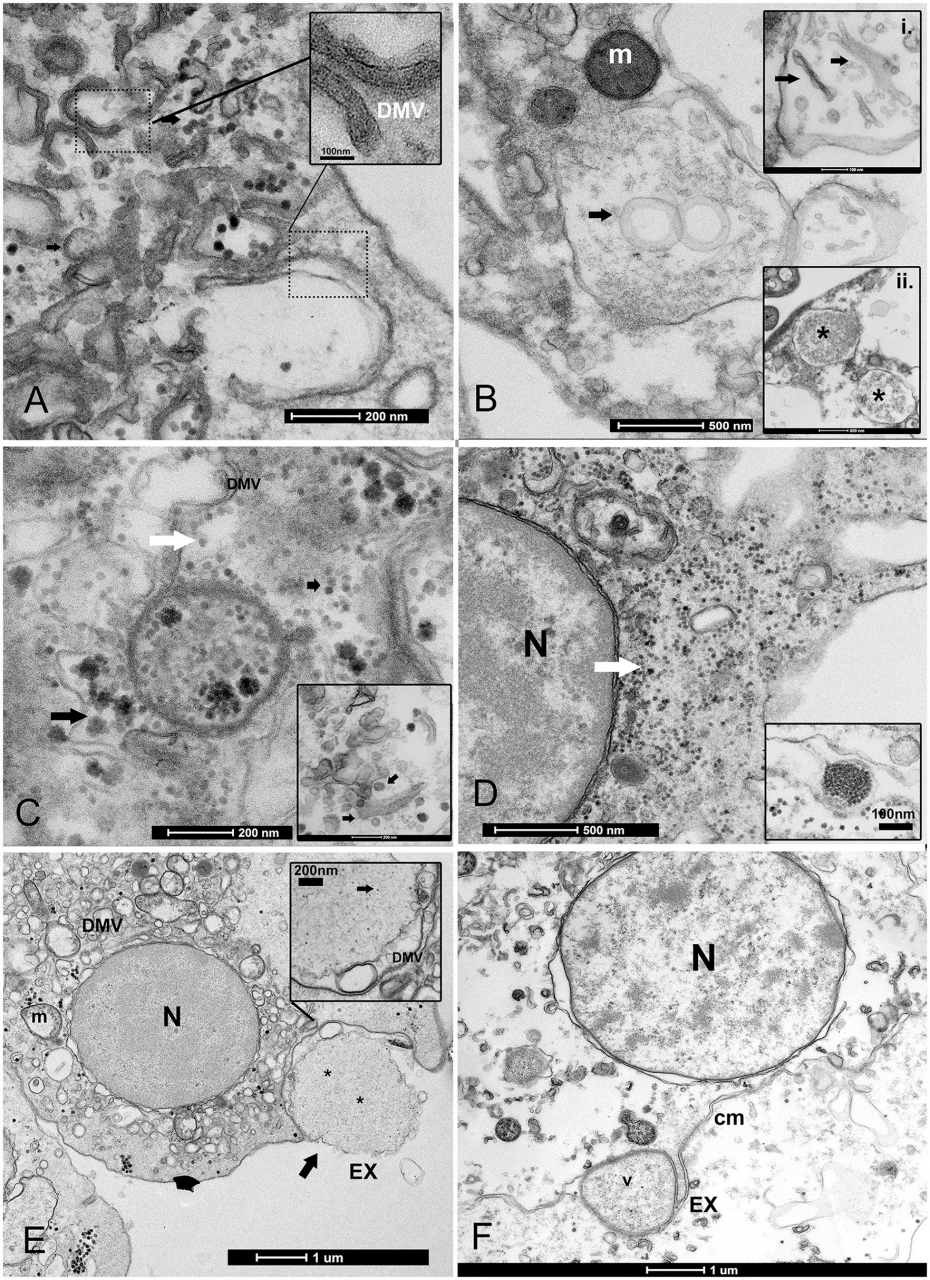
Biogenesis of double-membrane vesicles (DMVs) and exosome vesicles during PV replication. **(A)** Details of small and large DMVs with densely packed membranes that have an active role in viral RNA synthesis. **(B)** DMV biogenesis requires several membrane-remodeling steps: the biogenesis of DMVs can occur by the induction of positive membrane curvature through membrane pairing (inset i); these structures form cisternae that curve to finally seal and transform into a closed DMV and form ring-like vesicles, as reported in other picornaviruses where viral material can assemble (inset ii); **(C, D)** The virus replicates into factories through remodeled double membranes (inset), producing mature virions (white arrow) that accumulate in these large intracellular compartments from immature and forming ones (black arrow). **(D)** PV spreading into the granulocyte cytoplasm (white arrow); inset: grouped mature virions into vesicles. **(E, F)** Vesicles filled with viral particles (*) are released into the extracellular medium as exosomes (EX) after fusion with the cell membrane; viral egress involves the fusion of a double-membrane vesicle (V) with the cell membrane (CM); N, nucleus; m, mitochondria.

In all immune cells, aggregates of modified membranes occupied large areas of the perinuclear cytoplasm. Numerous membranous vesicles forming virions at various stages of self-assembly were visible. Vesicles in the cytoplasm were clearly associated with a dilated ER and nuclear membrane (Figures 3C, D). These vesicles had different dimensions and were constituted by double membranes, reported in the literature as DMVs or as autophagosome-like vesicles. The small vesicles measured 80–100 nm, whereas larger DMVs measured ~800–1,000 nm (Figures 3C–E). The presence of viral particles related to cytoplasmic vesicles, or mitochondrial membranes, was visible in all immune cells of *P. nobilis* (Figure 3F). Smaller vesicles were mostly used for virus genome replication at the cytoplasmic level, whereas larger DMVs supported a later step of virus production, specifically provirion maturation and the formation of complex factories. The DMVs displayed evident double or more complex membranes (Figure 4A). In earlier phases, the DMVs were filled by an unassembled viral amorphous and granular material; vesicles then showed one or two ring-like structures captured in the lumen vesicle, as also reported in other PVs (34) (Figure 4B). Viral replication factories are typically constituted by complex membrane remodeling emerging from DMVs formed by viral particles and proteins assembling mature virions, in some cases packed together. The virion is an icosahedral, non-enveloped, small (20 nm) particle with no discernible projections (Figures 4C, D).

Once the virus is assembled, viral release can occur through different mechanisms. Viruses can be released without lysis through so-called secretory autophagy, which occurs after the fusion of DMVs with the plasma membrane and are released as exosomes. Exosome typical dimensions ranged between 800 and 1,000 nm. Exosomes were secreted after their fusion with cell membrane vesicles and released as single-membraned virus-filled vesicles (Figures 4E, F). The four samples from Venice Lagoon, defined in the field as apparently healthy, displayed mature viral particles spreading into the cytoplasm (Figure 4D), small DMVs, and active factories.

**Figure 5:**
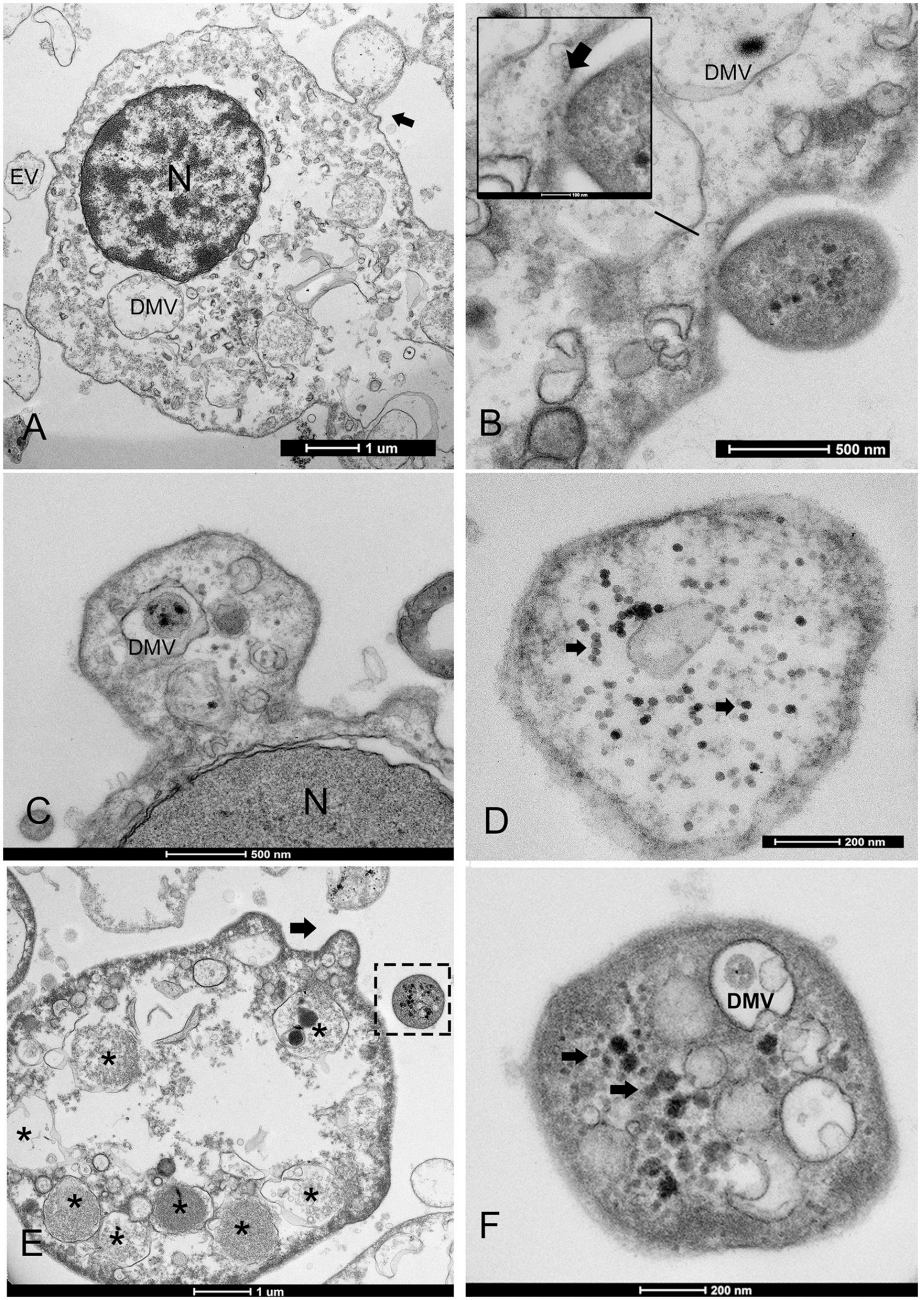
Extracellular vesicle (EV) formation during PV infection. **(A, B)** The EV lipid membrane mediates the release of virions through budding formation (arrow) with shedding microvesicles. **(B)** Details of budding from the cell membrane (arrow). **(C)** Hyaloncyte budding formation; **(D)** Vesicles might contain mature virions (arrows) or viral and/or host factor viral components (*) and induced/altered host factors **(E, F)**; N, nucleus; DMV, double-membrane vesicles within the EV.

**Figure 6:**
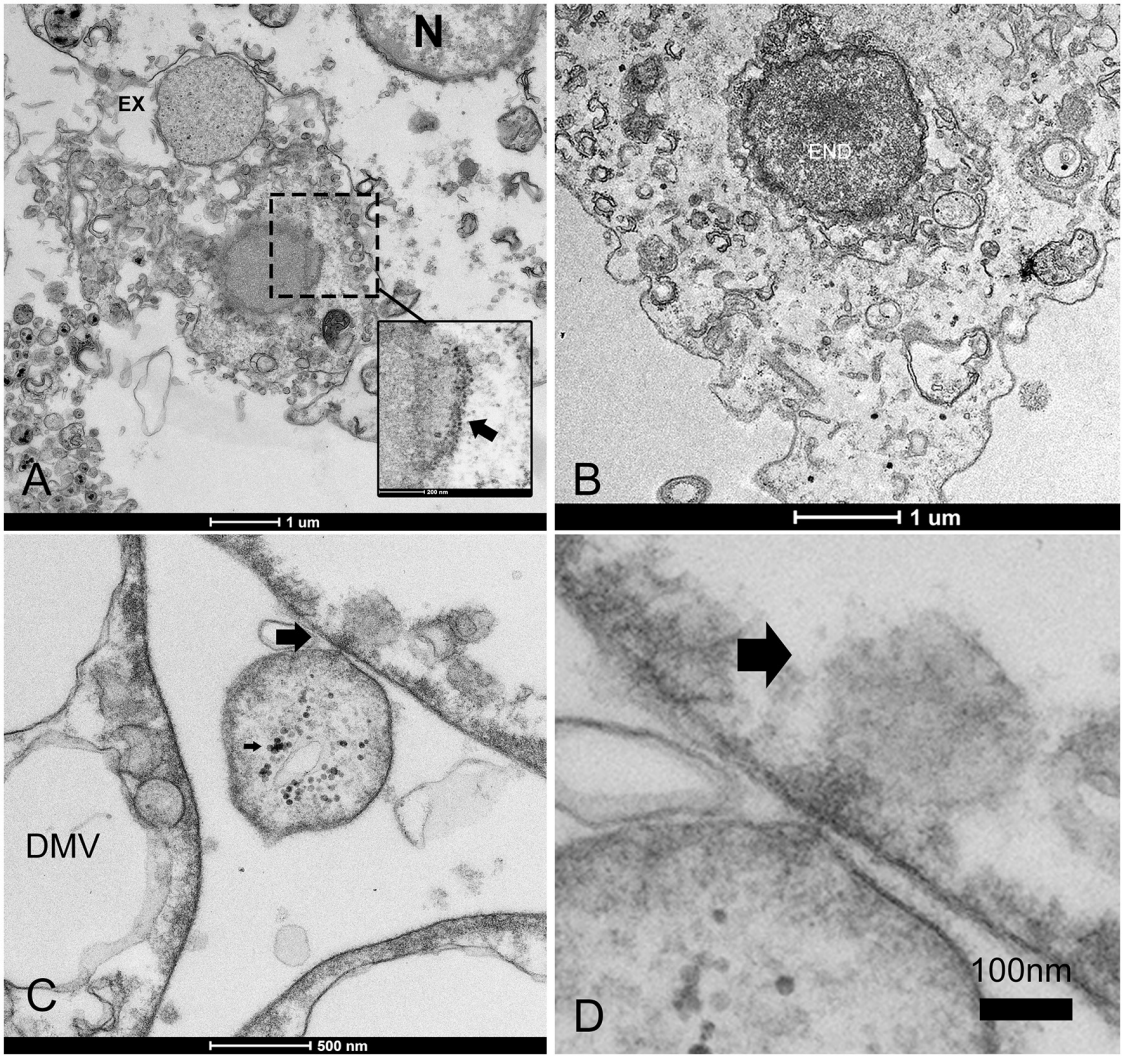
Infectious vesicles containing viral particles enter the cells. **(A, B)** Infective vesicles containing unassembled viral particles can enter through endocytosis, fusing with the cellular membrane and forming virions (inset, arrow) during the release of exosomes (EXs), which are then transported as late endosomes (ENDs) close to the nucleus **(B)**; **(C, D)** The vesicle can inject its content into other cells (arrows).

The predominant infective strategy observed in the PV of *P. nobilis*, typical for non-enveloped viruses, was a non-lytic “unconventional secretion” using extracellular vesicles (EVs) (Figure 5). PV-infected cells secreted EVs carrying viral RNA particles. Vesicles ranged in diameter from 100 to 700 nm. Virions may be released from cells by a budding process from both granulocytes and hyalinocytes (Figures 5A–C). Infective vesicles were observed carrying either mature viral particles (Figure 5D) or vesicle factories containing still immature virions and DMVs (Figures 5E, F). EVs may be taken up by recipient cells by endocytosis or fusion with the plasma membrane. The vesicle is internalized, via endocytosis, in an endocytic vacuole surrounded by DMVs to form mature viral particles (Figures 6A, B). Infective vesicles transporting mature virions and injecting their contents were also visible (Figures 6C, D).

**Figure 7:**
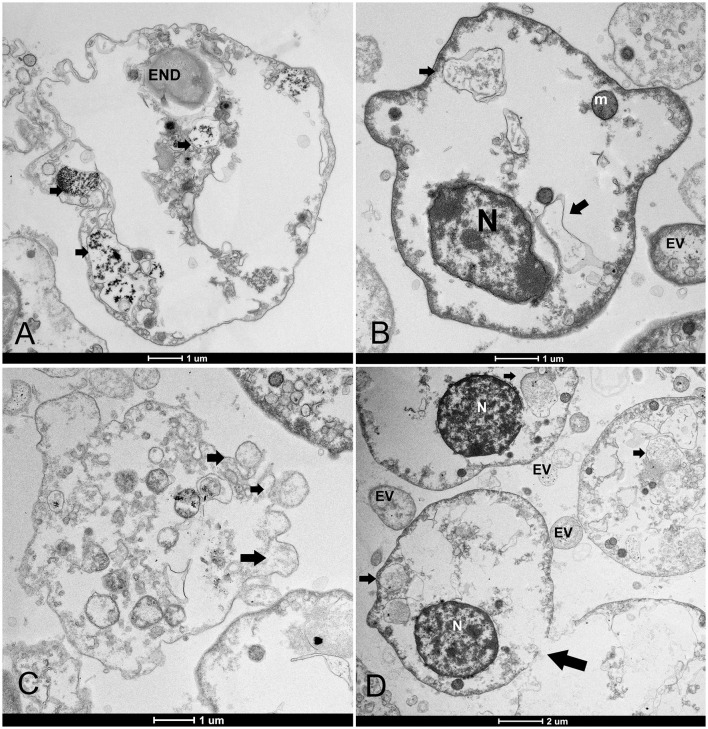
Hemocyte cell death. **(A, B)** Changes mainly include empty cytoplasm with residual vesicles (arrows) or endocytotic vesicles (ENDs) surrounded by extracellular vesicles (EVs); **(C)** blebbing of the plasma membrane and formation of apoptotic bodies (arrows) and loss of nuclei; **(D)** membrane rupture (arrows). N, nucleus; m, mitochondria.

Dead hemocytes were observed during infections. The highest number of damaged cells was observed in animals maintained in captivity at IMEDMAR-UCV and Murcia Aquarium, surrounded by an elevated number of EVs or containing endocytic vesicles. Damaged cells appeared empty, shrunk, contained few vesicles, and presented membrane rupture and apoptotic bodies (Figure 7).

### 3.2 Identification of RNA viruses in *P. nobilis* hemocytes

Sequencing of six stranded cDNA libraries retrieved from hemocyte samples of six *P. nobilis* using Illumina technology generated a total of 162,139,300 paired-end raw reads (Table 2), subsequently deposited in the NCBI Sequence Read Archive (SRA) under the BioProject accession PRJNA999583. After trimming and mapping against the *P. nobilis* genome, the number of unmapped paired reads was 33,097,808 (20.4%). These unmapped reads were used to assemble the viral RNA genomes present in the *P. nobilis* hemocytes.

BLASTX analysis of the 140,922 contigs assembled using Trinity resulted in 61,634 positive matches (43.7%, e-value < 10–20) against the viral protein database, encompassing DNA and RNA viruses as well as bacteriophages. Since this study was performed to identify RNA viruses in *P. nobilis* hemocytes, we focused specifically on this viral group using VirSorter2 and VirBot software tools. Both analyses identified the same set of five contigs, ranging from 5,210 to 8,876 nucleotides (nt) in length, all classified, with a score of 1 (the highest possible score with Virsorter2), into the realm *Riboviria*.

These five sequences exhibited the highest BLASTX matches with different *Riboviria* polyproteins 1 and 2 (Table 5), mainly of *Picornavirales*. Notably, two of these contigs (TRINITY_DN35348_c1_g1 and TRINITY_DN9545_c0_g1) displayed the highest number of mapped reads, particularly in the Trab samples (Site 1, Trabucador), indicating their abundance among the five identified viruses. Additionally, during the global BLASTX analysis, we discovered the contig TRINITY_DN58105_c0_g1_i1 (841 nt), which matched the predicted replication-associated protein of *Chaetoceros tenuissimus* RNA virus type II (BAP99820.1). Manual overlap with TRINITY_DN35348_c1_g1 (5,210 nt, matched with the predicted structural protein of *Chaetoceros tenuissimus* RNA virus type II, BAP99821.1) resulted in a 6,023 nt consensus sequence.

**Table 5:** Assembled contigs classified into the realm *Riboviria* by VirSorter2 and VirBot analysis, the results of their annotation through BLASTX vs. the viral protein database, and read abundance in the different noble pen shell *Pinna nobilis* samples expressed as raw counts.

**Trinity contig name**	**Length (nt)**	**Best BLASTX hit**	**Bar1**	**Bar2**	**Trab8a**	**Trab8b**	**XIR2a**	**XIR5**
TRINITY_DN35348_c1_g1	5,210	Predicted structural protein [*Chaetoceros tenuissimus* RNA virus type II]	102	57	6,544	4,113	255	89
		BAP99821.1						
TRINITY_DN9545_c0_g1	8,505	Polyprotein [*Picornavirales* sp.]	13	3,755	3,675	4,432	19	11
		UNY42036.1						
TRINITY_DN33054_c0_g1	8,876	Putative non-structural protein [*Picornavirales* N_OV_064]	3	364	406	509	2	1
		putative structural protein, partial [*Picornavirales* N_OV_064] ASG92536.1						
TRINITY_DN33329_c0_g1	5,810	P1–P2 fusion protein [Pepo aphid-borne yellows virus] ANF99514.1	1.05	411.89	345.38	413.98	2.09	0
TRINITY_DN37302_c0_g1	8,567	Polyprotein [marine RNA virus BC-7] AYD68770.1	0	415	386	466	5	0

**Figure 8:**
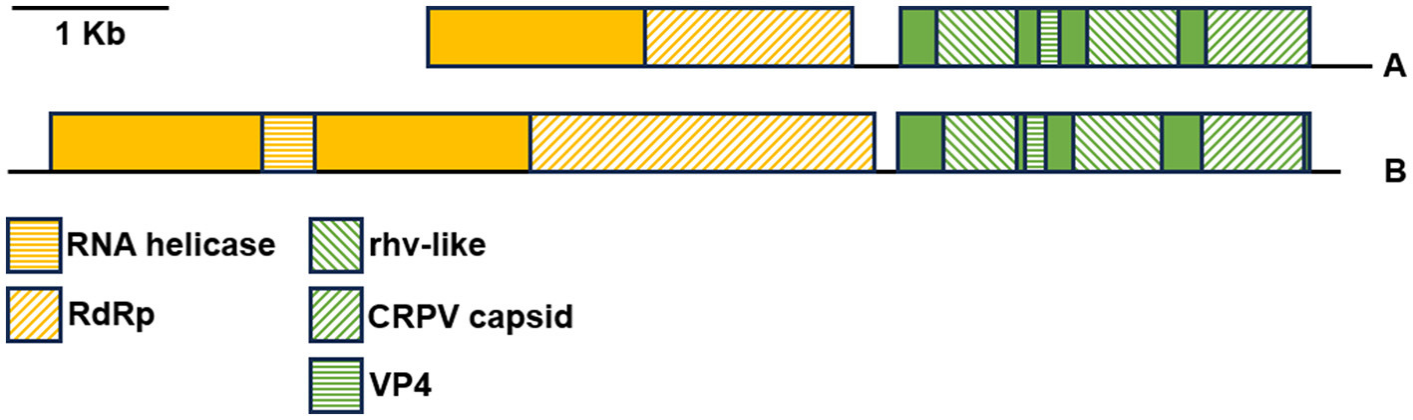
Genomic organization of the most abundant RNA viruses identified in the noble pen shell *Pinna nobilis* hemocytes. The colored boxes represent the ORFs of genes 1 (yellow) and 2 (green) encoding viral polyproteins 1 and 2, respectively. The different colored motifs represent the regions encoding specific viral proteins. **(A)** Trinity-assembled contigs DN35348_c1_g1 and DN58105_c0_g1, 5′-end partial (accession number OR448788); **(B)** Trinity-assembled contig DN9545_c0_g1 (accession number OR448789).

Figure 8 presents the genomic organization of the two most prevalent viruses identified herein. The sequence consensus_DN35348_c1_g1_DN58105_c0_g1 **(A)** is partial, lacking the complete 5′-end, while the DN9545_c0_g1 sequence **(B)** is complete. These two sequences were deposited in GenBank under accessions OR448788 and OR448789. The ORFfinder analysis identified two large ORFs encoding two viral polyproteins in both sequences. Within polyprotein 1, there were conserved domains of RdRp and RNA helicase (the latter was detected only in sequence DN9545). In contrast, structural polyproteins 2 have two rhv-like domains (picornavirus capsid protein domain-like), a CRPV capsid domain (a family of capsid proteins found in positive stranded ssRNA viruses), and a short VP4 domain (a family of minor capsid proteins located within the viral capsid at the interface between the external protein shell and packaged RNA genome). The presence of two large ORFs encoding these specific domains suggests that these viral sequences belong to the *Picornavirales* order.

The genomic organization of the remaining three viruses identified in *P. nobilis* hemocytes found herein (DN33054, DN37302, and DN33329) was deposited in GenBank under accession numbers OR448790, OR448791, and OR448792, respectively (Supplementary Figure 1). Similar to the two most abundant viruses, the genomic organization of DN33054 and DN37302 presented two large ORFs, with ORF1 encoding RdRp and RNA helicase and ORF2 encoding two rhv-like domains, a CRPV capsid domain and a short VP4 domain. In contrast, the genomic organization of DN33329 was quite different, with three partially overlapping main ORFs encoding a peptidase and RdRp, similar to the structure of the *Sobelivirales* order.

**Figure 9:**
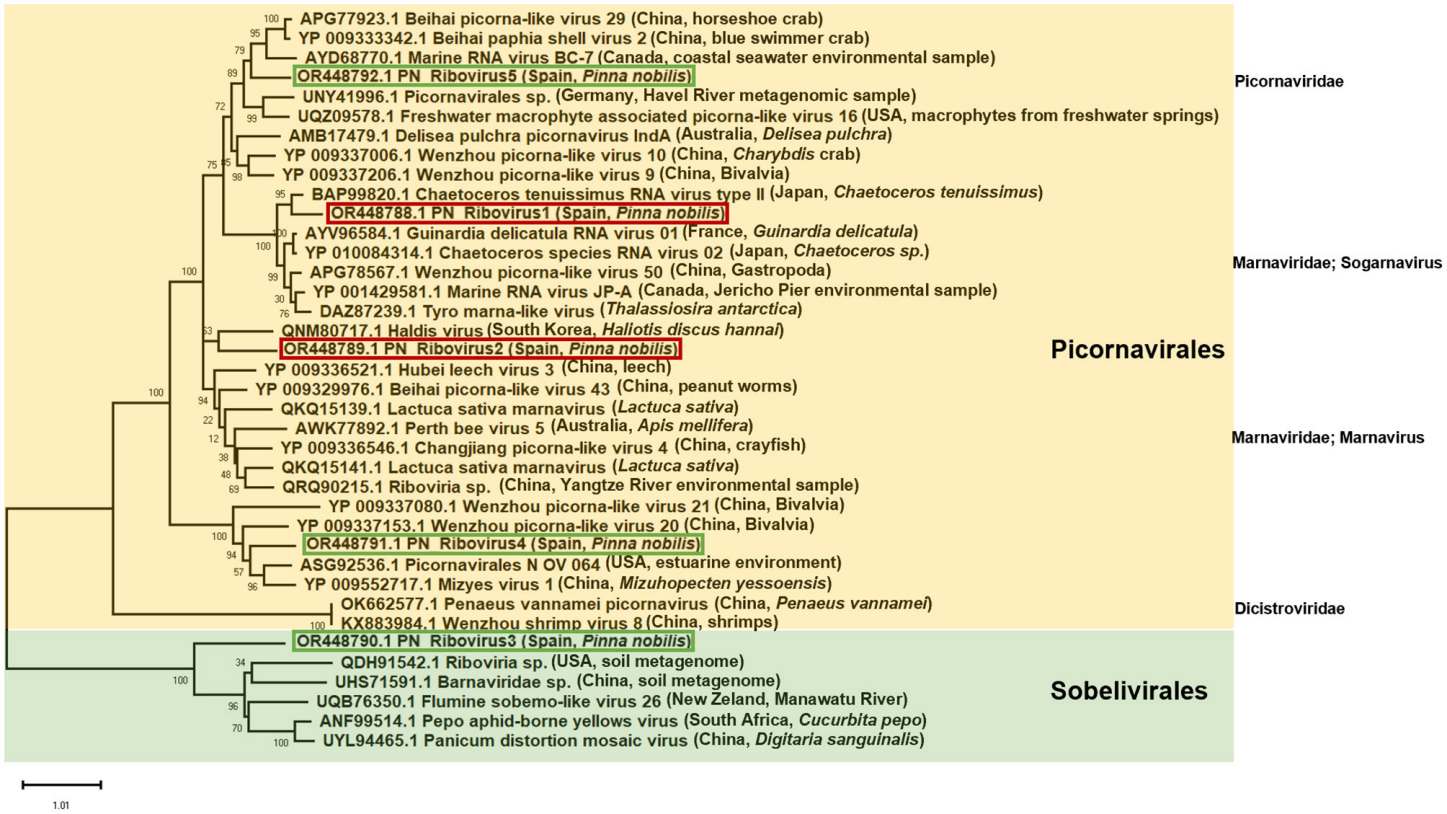
Maximum likelihood tree constructed using the amino acid alignment of the conserved region of the RpRd of the RNA viruses identified in the noble pen shell *Pinna nobilis* hemocytes and other RpRd proteins downloaded from GenBank (total 38 amino acid sequences). The tree with the highest log likelihood (−16,083.36) is shown. The tree is drawn to scale, with branch lengths measured in the number of substitutions per site. The red and green boxes highlight the most and least abundant RNA viruses identified, respectively. The bootstrap percentages inferred from 500 replicates are shown next to the branches. When available, the geographic origin and host of the samples are indicated within parentheses.

The maximum likelihood tree was generated based on the alignment of the conserved region of the RdRp proteins of the viruses identified in the *P. nobilis* hemocytes with homologous sequences downloaded from GenBank (Figure 9). Almost all of the RdRp sequences downloaded from GenBank belonged to *Picornavirales* from marine or freshwater environmental samples. A restricted number of sequences belonged to *Sobelivirales*, and two of them (*Penaeus vannamei* picornavirus and Wenzhou shrimp virus 8) to *Dicistoviridae*, which are associated with mollusk and crustacean diseases (27, 51). Among the viral sequences identified in *P. nobilis* hemocytes, DN33329 clustered with those in the order *Sobelivirales*, along with viruses from rivers, soil, and plants, agreeing with the genomic organization previously described (Supplementary Figure 1). Additionally, the remaining four sequences from *P. nobilis* belonged to the order *Picornavirales*, as expected from their genomic organization; among them, the two most abundant were clustered within the family *Marnaviridae*.

In terms of ultrastructure, both samples from the Trabucador area (Trab8a and Trab8b) that had the highest number of mapped viral reads displayed numerous cytoplasmic viral factories and extracellular infective vesicles spreading into the hemolymph, suggesting an active infective phase.

### 3.3 Detection of RNA virus in *P. nobilis* hemocytes by quantitative real-time PCR

**Figure 10:**
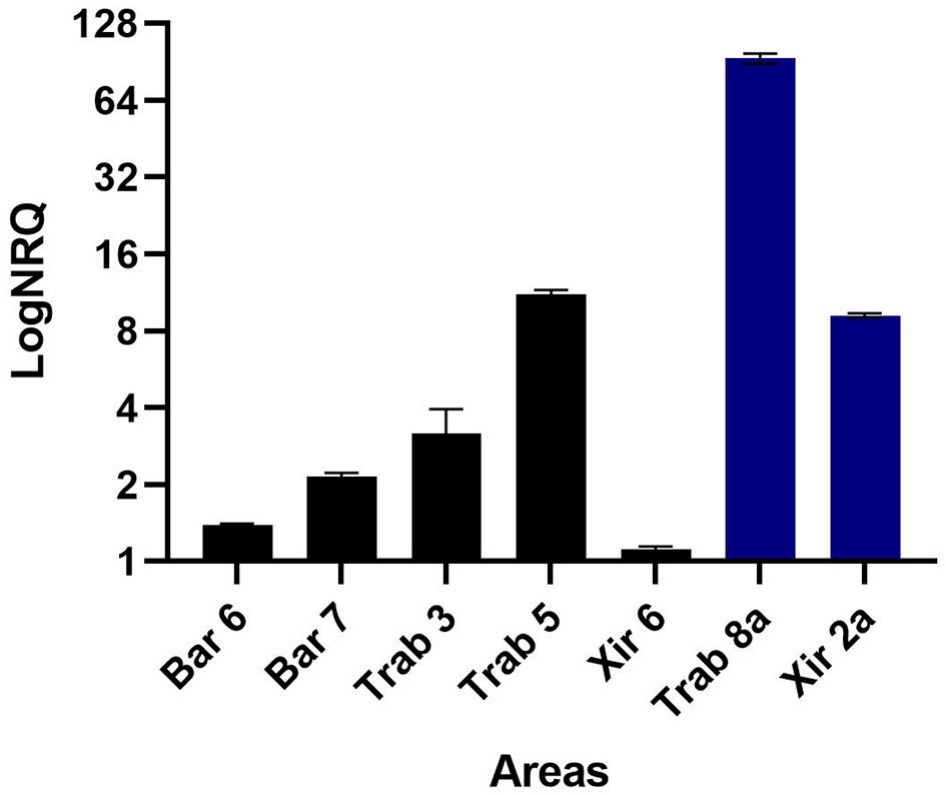
Results of the qPCR experiment conducted on the cDNA of the noble pen shell *Pinna nobilis* hemocytes collected in different areas using primer pairs specific for the *in silico* assembled Sogarnavirus (accession number OR448788). Bars represent the logarithm of the mean normalized relative quantification ± SEM using *P. nobilis* actin as an endogenous gene. The blue bars represent the samples used for the *in silico* RNA-seq experiment.

The quantitative real-time PCR experiments conducted on the cDNA of the *P. nobilis* hemocytes collected at different sampling points in Catalonia were positive for the primer pair PNChetoF/R designed on the reconstructed DN35348_DN58105 sequence, similar to a *Chaetoceros tenuissimus* RNA virus type II. These results confirm the *in silico* analysis of viral read abundance (Table 5), with Trab8a and XIR2a showing the highest infection level and XIR5 showing a low infection level (Figure 10). With the same primer pair, viral RNA was detected in all other examined samples, presenting variable infection levels. The primer pair PNPicorF/R designed for the sequence DN9545 did not provide a detectable amplification signal.

## 4 Discussion

This study reports the discovery of a picornavirus affecting *P. nobilis* hemocytes in Spain and Italy during MMEs (1, 4, 52). Previous studies investigating the etiological agent involved in MMEs showed a complex pattern: disease diagnosis repeatedly confirmed the simultaneous presence of several agents, such as *Haplosporidium pinnae* and *Mycobacterium* spp. (1, 4, 13), *Vibrio mediterranei* (53), and in some cases *Perkinsus* spp., suggesting that a common primary cause, not yet identified, could be linked to these phenomena (4). Based on our observations, this picornavirus could be the underlying cause of these events, as the virus was actively replicating and infecting *P. nobilis* shell immune cells in 100% of the examined residual populations in Italy and Spain from 2021 to 2023. In fact, the detection of this virus in the same populations over a 2-year period suggests that animals can maintain persistent infections until the disease becomes chronic, weakening them and making them susceptible to opportunistic infections, as also reported in other immunosuppressive viruses (54). This could explain the apparent resistance of the residual individuals who are slowly dying in all areas. The affected animals remain apparently healthy for months to years before the immune system begins to collapse, as reported by many scientists (3, 11, 52).

In this study, the observed picornavirus affecting *P. nobilis* in Italy and Spain used granulocytes and hyalinocytes as vessels for dissemination, replication, and long-term persistence, interfering with hemocyte immune abilities, as observed in other picornaviruses (55). Multiple known immunoregulatory mechanisms play a role in persistent viral infections, resulting in immunosuppression (56). Immunodeficiency viruses have been reported worldwide in felid, bovine (dairy cattle), and primate (chimpanzee) species and have been observed actively replicating in blood and lymphoid tissues (57). As a result of complete or unsuccessful viral replication, immunodeficiency viruses may functionally lyse or impair immune cells such as lymphocytes, as observed in parvovirus infections of B lymphocytes, in infectious mononucleosis due to Epstein–Barr virus, and in CD4^+^ T lymphocytes and macrophages in AIDs caused by HIV lentivirus (58, 59). In other cases, viruses can infect, and damage cells involved in phagocytosis, such as macrophages, as seen in influenza virus and dengue virus infections (60, 61). Similarly, PV can block the production of type I interferon (IFN), leading to T-cell depletion, or modulate immune cell apoptosis and autophagy mechanisms (62–64). In bivalves, the primary role of hemocytes is pathogen killing and elimination through phagocytosis, encapsulation, production of cytotoxic molecules and antimicrobial peptides, and secretion of inflammatory cytokines (65–68). A morpho-functional description of *P. nobilis* hemocytes was performed prior to the mortality events in 2015, showing that healthy hemocytes were able to conduct phagocytosis (69). In our study, *P. nobilis* hemocytes from individuals sampled in Spain and Italy displayed damage in mitochondria, ER, and other organelles, which may have affected their regular immune function (70). This is also true considering that a preliminary study performed in *P. nobilis* shell hemocytes from the same areas in 2021–2022 already revealed strong immunodepression, represented by a decreased immune cell response to pathogenic stimuli following an *in vivo* challenge (11).

Immunosuppression is necessary for PVs to use cell components to promote infection (55). PVs have evolved the ability to usurp infected cells' endogenous functions, inducing (endo)membrane rearrangements that are referred to as viral replication organelles (71). Recent work revealed that picornaviruses pack multiple viral particles into a cellular microvesicle to promote the spread of several heterogeneous virions at a time (72). In this context, the production of EVs and endocytic vesicles plays an important role in the pathogenesis and establishment of viral genome persistence and latency; moreover, viruses that establish chronic infections have been shown to modulate the production and content of more infectious EVs, increasing their infective potential (73). Animals maintained in captivity in this study displayed a high number of infective vesicles and dead cells, which was also related to hemocytopenia. Persistent PV infection can induce lymphopenia in humans and animals, such as cows, cats, and birds, as a direct result of viral infection or indirectly through cytokine induction (55, 74). Along with other diagnostic indices, decreased THC is a critical condition in the progression of an infection or a disease in invertebrates (75). Heamocytopenia has been observed during chronic viral infections, such as abalone ganglioneuritis (76), and during bacterial and parasitic diseases in other mollusk species (77, 78). Typically, persistent infections bring a relatively small pool of hemocytes into the hemolymph sinuses and perivisceral beds, and such sequestration into tissues may lead to a dramatic decrease in hemocyte numbers (79). Before the observation of mass mortality in the Mediterranean, Matozzo et al. (69) reported a higher number of circulating hemocytes (5 × 10^5^ cells) in comparison to that in the current Spanish wild and captive populations. Based on our results, this finding could indicate an over-time immune cell decrease due to infection and a worsened situation for animals maintained in tanks. Apart from hemocyte sequestration into infected tissues, such hemocytopenia is also possibly caused by apoptosis due to hemocyte infection (80). This host-initiated process of programmed cell death is essential for normal cell turnover and functions as an antiviral mechanism to limit the spread of a virus (81, 82).

In the present study, we were able to assemble different *Riboviria* sequences of the orders *Picornavirales* and *Sobelivirales*, with NGS Illumina sequencing obtaining nearly five different genomes accompanied by phylogenetic analysis. Our findings support previous observations that bivalve and other invertebrate viromes harbor a broad diversity of viruses of the order *Picornavirales* (25, 83), matching the ultrastructural features of hemocyte PV infection. Picornaviruses are components of the virome associated with filter-feeding organisms under normal physiological conditions, as reported in metatranscriptomic studies in the absence of detectable disease (84). Among the five assembled *Riboviria*, the two most abundant sequences are clustered with the *Picornavirales* group within the family *Marnaviridae*, which comprises small non-enveloped viruses that infect various photosynthetic marine protists (85) and have also been reported in diatom populations (86). The most abundant reads placed the hemocyte PV close to a virus isolated in the marine diatom *Chaetoceros tenuissimus*, named *C. tenuissimus* RNA virus type II (86), belonging to the genus *Sogarnavirus*, while the second most abundant PV was identified as *Picornavirus*, belonging to the genus *Marnavirus*.

Microalgal cells infected by *Marnaviridae*, genus *Sogarnavirus*, can display extensive cytopathic effects with ultrastructural changes, including swelling of the endoplasmic reticulum, vacuolation, and disintegration of the cytoplasm (86, 87), as reported in our study. To the best of our knowledge, this is the first report of a sogarnavirus affecting an animal species. To confirm the NGS analysis, qPCR was performed to amplify the most represented *Sogarnavirus*. The primer pairs designed on the sequence of the *P. nobilis* picornavirus similar to the *C. tenuissimus* RNA virus type II were positive in the analyzed samples, while no amplification was obtained with the other primer pairs. These results may not be conclusive and need to be confirmed with other analyses.

In this study, both apparently healthy and sick animals showed ultrastructural features of viral infection. In confined waters across Mediterranean regions, such as lagoons, MMEs started later (52). For instance, in Venice Lagoon, mortality episodes began in 2020, which is after the *P. nobilis* populations started dying off of the Ebro Delta, which started in 2018 (4). The viral infection could have originated first in Spanish waters, years before mortalities became evident, subsequently reaching other areas, until animals presented an acquired immunodeficiency (i.e., low count of hemocytes), as occurs with other immunodeficiency viruses. The disease, made up of periods of remission followed by disease progression over time, ends up gradually weakening the populations that start to display symptoms, leading to population decline. These individuals are exposed to a wide variety of bacterial and parasitic opportunistic infections, resulting in an unfavorable situation. In the Venice Lagoon, in the area where animals appeared apparently healthy in May 2023, remarkable mortality was observed 2 months later. In the four analyzed individuals, all immune cells presented actively replicating virus. This means that infection can be subclinical until very late in the process, which is when an opportunistic pathogen is more likely to take advantage of the situation.

As previously reported, recent findings suggest a more complex scenario than the one originally proposed in the first descriptions of *P. nobilis* MMEs (1, 4, 13). Diseases with complex etiology are influenced by various interacting drivers and frequently remain difficult to characterize. Indeed, this viral disease represents an epidemiological threat of unknown proportions for marine animal populations. Further studies are needed to determine the ecological importance of PV as an agent affecting the dynamics of *P. nobilis* MMEs in the analyzed areas and in other natural environments.

## 5 Conclusion

Few viruses, mainly those belonging to the families of *Alloherpesviridae* and *Iridoviridae*, have been associated with disease outbreaks and mortality in bivalves. Nevertheless, picornavirus-like particles have also been reported during disease outcomes (88–90). Here, we report the first description of an immunodeficiency virus in bivalves affecting *P. nobilis* hemocytes. Immune defense is a key determinant of fitness, underlying the capacity of animals to resist or tolerate potential infections. Immune function is not static and can be suppressed, depressed, or stimulated by exposure to environmental abiotic factors, food availability, and pathogens. Alteration of this response can directly affect population dynamics and survival. Recently, the emergence and spread of new and existing pathogens have posed a significant risk not only to human life and animal health but also to the conservation of wildlife. Future studies are needed to establish a clear and significant correlation between the genome of the picornaviruses assembled in *P. nobilis* hemocytes and the *P. nobilis* MMEs. First, the full length of the partial PV sequence assembled *in vitro* should be obtained using a 5′RACE approach. Second, viral isolation and cultivation attempts are needed to define virus characteristics. Finally, a fast and reliable diagnostic test (e.g., based on qPCR) should be developed to rapidly check the infection level of the surviving *P. nobilis* populations in other geographic areas. Virus presence should also be evaluated in the environment (water, plankton, sediments) and surrounding animals, which could be potential virus reservoirs.

## Data availability statement

The original contributions presented in the study are publicly available. This data can be found here: https://www.ncbi.nlm.nih.gov/bioproject; PRJNA999583.

## Ethics statement

The animal study was approved by MATTM Ministero dell'ambiente e della sicurezza energetica. The study was conducted in accordance with the local legislation and institutional requirements.

## Author contributions

FC: Conceptualization, Data curation, Formal analysis, Funding acquisition, Investigation, Methodology, Supervision, Validation, Writing—original draft, Writing—review & editing. PP: Investigation, Resources, Writing—review & editing. GD: Writing—review & editing. DP: Writing—review & editing. GV: Methodology, Writing—review & editing. JG-M: Resources, Writing—review & editing. JT-M: Resources, Writing—review & editing. EC: Resources, Writing—review & editing. FG-C: Resources, Writing—review & editing. MS: Writing—review & editing. SA: Data curation, Formal analysis, Software, Supervision, Writing—original draft, Writing—review & editing.

## Supplementary material

Supplementary Figure 1 Genomic organization of the less abundant RNA viruses identified in the noble pen shell *Pinna nobilis* hemocytes. The colored boxes represent the ORFs of genes 1 (yellow) and 2 (green) encoding viral polyproteins 1 and 2, respectively. **(A)** Trinity-assembled contig DN33329_c0_g1 (accession number OR448790); **(B)** Trinity-assembled contig DN33054_c0_g1 (accession number OR448791); **(C)** Trinity-assembled contig DN37302_c0_g1 (accession number OR448792).
