# Supplementary figures and images for: A widespread picornavirus affects the hemocytes of the noble pen shell (Pinna nobilis), leading to its immunosuppression
Source: Front Vet Sci. 2023 Dec 13;10:1273521. doi: 10.3389/fvets.2023.1273521 (PMC10758234; doi:10.3389/fvets.2023.1273521)

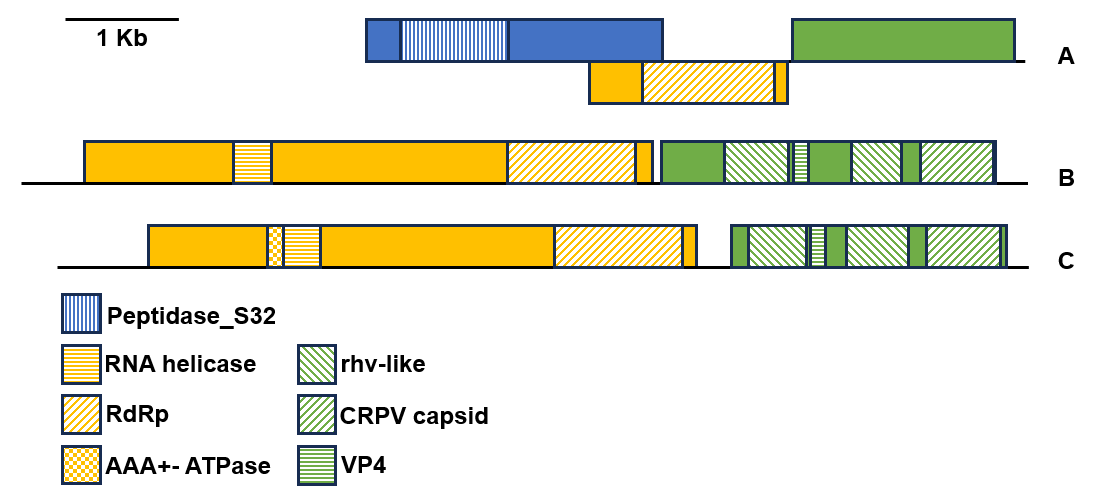

Supplement: Supplementary Figure 1 — Genomic organization of the less abundant RNA viruses identified in the noble pen shell Pinna nobilis hemocytes. The colored boxes represent the ORFs of genes 1 (yellow) and 2 (green) encoding viral polyproteins 1 and 2, respectively. (A) Trinity-assembled contig DN33329_c0_g1 (accession number OR448790); (B) Trinity-assembled contig DN33054_c0_g1 (accession number OR448791); (C) Trinity-assembled contig DN37302_c0_g1 (accession number OR448792). [file Image_1.TIF]
